# Synaptotagmin I Regulates Patterned Spontaneous Activity in the Developing Rat Retina via Calcium Binding to the C2AB Domains

**DOI:** 10.1371/journal.pone.0047465

**Published:** 2012-10-16

**Authors:** Chung-Wei Chiang, Yu-Chieh Chen, Juu-Chin Lu, Yu-Tien Hsiao, Che-Wei Chang, Pin-Chien Huang, Yu-Tzu Chang, Payne Y. Chang, Chih-Tien Wang

**Affiliations:** 1 Institute of Molecular and Cellular Biology, National Taiwan University, Taipei, Taiwan; 2 Department of Life Science, National Taiwan University, Taipei, Taiwan; 3 Neurobiology and Cognitive Science Center, National Taiwan University, Taipei, Taiwan; 4 Genome and Systems Biology Degree Program, National Taiwan University, Taipei, Taiwan; 5 Department of Physiology and Pharmacology, College of Medicine, Chang Gung University, Kwei-Shan, Tao-Yuan, Taiwan; 6 Center for Learning and Memory, University of Texas at Austin, Austin, Texas, United States of America; Virginia Commonwealth University Medical Center, United States of America

## Abstract

**Background:**

In neonatal binocular animals, the developing retina displays patterned spontaneous activity termed retinal waves, which are initiated by a single class of interneurons (starburst amacrine cells, SACs) that release neurotransmitters. Although SACs are shown to regulate wave dynamics, little is known regarding how altering the proteins involved in neurotransmitter release may affect wave dynamics. Synaptotagmin (Syt) family harbors two Ca^2+^-binding domains (C2A and C2B) which serve as Ca^2+^ sensors in neurotransmitter release. However, it remains unclear whether SACs express any specific Syt isoform mediating retinal waves. Moreover, it is unknown how Ca^2+^ binding to C2A and C2B of Syt affects wave dynamics. Here, we investigated the expression of Syt I in the neonatal rat retina and examined the roles of C2A and C2B in regulating wave dynamics.

**Methodology/Principal Findings:**

Immunostaining and confocal microscopy showed that Syt I was expressed in neonatal rat SACs and cholinergic synapses, consistent with its potential role as a Ca^2+^ sensor mediating retinal waves. By combining a horizontal electroporation strategy with the SAC-specific promoter, we specifically expressed Syt I mutants with weakened Ca^2+^-binding ability in C2A or C2B in SACs. Subsequent live Ca^2+^ imaging was used to monitor the effects of these molecular perturbations on wave-associated spontaneous Ca^2+^ transients. We found that targeted expression of Syt I C2A or C2B mutants in SACs significantly reduced the frequency, duration, and amplitude of wave-associated Ca^2+^ transients, suggesting that both C2 domains regulate wave temporal properties. In contrast, these C2 mutants had relatively minor effects on pairwise correlations over distance for wave-associated Ca^2+^ transients.

**Conclusions/Significance:**

Through Ca^2+^ binding to C2A or C2B, the Ca^2+^ sensor Syt I in SACs may regulate patterned spontaneous activity to shape network activity during development. Hence, modulating the releasing machinery in presynaptic neurons (SACs) alters wave dynamics.

## Introduction

Binocular visual circuit refinement requires a robust patterned spontaneous activity in the developing retina (i.e. retinal waves). Retinal waves are spontaneous, correlated action potentials propagating through the retinal ganglion cell (RGC) layer during a developmental critical period prior to visual experience. In particular, retinal waves are mediated via synaptic transmission with a periodicity on the order of minutes. In the developing mammalian retina, three different stages of retinal waves have been classified, of which the stage-II waves are the best studied [1], [2], [3]. The stage-II waves during postnatal days (P0–P9) are initiated by a subset of starburst amacrine cells (SACs) releasing neurotransmitters acetylcholine (ACh) and γ-aminobutyric acid (GABA) onto neighboring SACs and RGCs, allowing depolarizations to propagate across the RGC layer [4], [5], [6], [7]. Correlated depolarizations further induce Ca^2+^ influx. Accordingly, the intracellular Ca^2+^ concentrations exhibit spontaneous and correlated increases, producing wave-like Ca^2+^ oscillations across the RGC layer [8], [9], [10]. Imaging spontaneous and correlated Ca^2+^ transients in RGCs has been widely used to elucidate the synaptic mechanisms underlying retinal waves [2], [3].

Synaptic transmission is initiated by neurotransmitter release via Ca^2+^-regulated exocytosis. Members of the Ca^2+^ sensor protein family synaptotagmin (Syt) transduce the Ca^2+^ signal to trigger fusion between vesicle and plasma membranes [11], [12], [13]. Syts can bind up to five Ca^2+^ through their two Ca^2+^-binding C2 domains (C2A and C2B), with three binding to C2A and two binding to C2B [14], [15]. Upon Ca^2+^ binding, Syts interact with distinct effectors, such as lipids, SNARE (***s***oluble ***N***SF ***a***ttachment protein ***re***ceptor) proteins, assembled SNARE complex, and other Syt molecules to trigger exocytosis [15], [16], [17].

Previous studies revealed that preferential Ca^2+^ binding to distinct C2 domains in Syt controls the choice of exocytotic modes [18], [19], regulates the kinetics of exocytosis, and modulates the dynamics of exocytotic fusion pores [20], [21]. In particular, weakened Ca^2+^ binding to C2A reduces full fusion by limiting fusion pore dilation and preventing release of some vesicle content. By contrast, weakened Ca^2+^ binding to C2B abolishes both full fusion and kiss-and-run [18], [19], [21]. Detailed kinetic analysis has resolved two separate sequential steps in regulated exocytosis, fusion pore opening and dilation, which are differentially controlled by Ca^2+^ binding to C2A or C2B in Syt I [21], [22]. As a result, Ca^2+^ binding to various C2 domains of Syt confers a repertoire of functions in controlling neurotransmitter release and synaptic transmission [17], [18], [21], [23], [24], [25], [26], [27]. However, the impact of these molecular signaling processes on large-scale, repetitive synaptic circuit activity has not been investigated.

Many studies have characterized the role of SACs in regulating wave dynamics during the stage-II period. However, the release mechanism in SACs has not been well understood. Immunolabeling and confocal microscopy suggested that several SNARE proteins are expressed in SACs, such as syntaxin 1 and 2 [28], synaptobrevin 2 [29] and SNAP-25 [30]. However, their functional roles in SACs have not been characterized. Classic physiological experiments showed that perinatal rabbit SACs demonstrate oscillations of Ca^2+^ spikes and Ca^2+^-dependent afterhypolarizations [6], suggesting release from SACs is Ca^2+^-dependent. Borges et al. reported that a small number of primed vesicles, very likely one per release site, can be quickly exocytosed upon Ca^2+^ influx into SACs [31]. These findings point out the possibility that oscillations of Ca^2+^ influx into SACs may cause Ca^2+^ binding to its sensor Syt to drive exocytosis during stage-II waves.

Although the human genome encodes more than seventeen Syt isoforms [32], the Syt isoform responsible for the initiation of stage-II waves remains completely unknown. Moreover, it is unclear how the C2AB domains of Syt regulate stage-II waves by controlling specific characteristics of retinal waves. Here we used immunofluorescence, molecular perturbation and live Ca^2+^ imaging to investigate the expression of Syt I in SACs and determine the roles of C2AB domains in regulating stage-II waves during the development of neural circuits.

## Results

### Syt I is Strongly Expressed in Cholinergic Synapses of the Developing Retina

**Figure 1:**
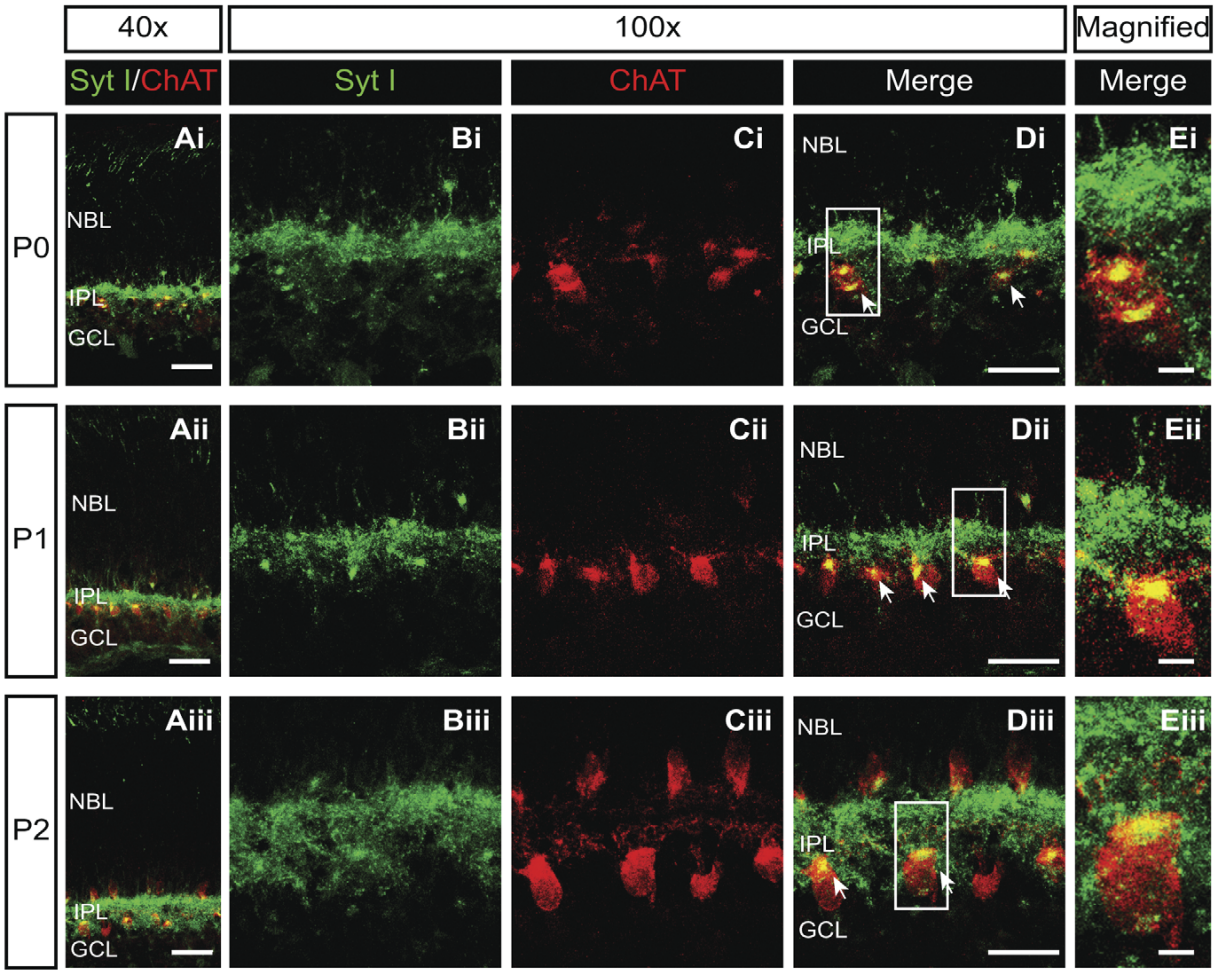
Syt I is strongly expressed in neonatal SACs and IPL. Ai–Aiii. Merged images of Syt I (green) and ChAT (red) staining in retinal cross-sections from P0–P2 rats. The colocalization of Syt I and ChAT labeling was found in the IPL (yellow). Scale bars, 25 µm. ChAT, choline acetyltransferase. NBL, neuroblast layer; IPL, inner plexiform layer; GCL, ganglion cell layer. Bi–Biii. Immunofluorescence labeling of Syt I (green) in the IPL of retinal cross-sections from P0–P2 rats. Ci–Ciii. Immunofluorescence staining of ChAT (red) labeling SACs and the IPL in retinal cross-sections from P0–P2 rats. Di–Diii. Merged images of Syt I and ChAT staining in the same retinal cross-sections. The Syt I and ChAT labeling was colocalized to the SAC somata (yellow) as indicated by arrows. Scale bars for B-D, 50 µm. Ei–Eiii. The high magnification for merged images of Syt I and ChAT staining in the boxes of Di–Diii. Scale bars for E, 5 µm.

Syt I is the major Syt isoform found in many parts of the nervous system [15]. However, the expression of Syt I in the developing SACs, which release neurotransmitters to initiate stage-II waves, remains to be determined. We thus examined if Syt I is expressed in SACs during stage-II waves. In P0–P2 rat retinal cross-sections, double immunolabeling for Syt I and choline acetyltransferase (ChAT, the SAC marker) showed that Syt I was mainly localized to the inner plexiform layer (IPL) surrounding the SACs (Fig. 1). The high-magnification image from the IPL indicated that Syt I was also localized to the somata of SACs (Fig. 1Ei–iii, yellow), suggesting that Syt I is expressed in the cholinergic synapses originating from SACs. Thus, Syt I is located where it can serve as a Ca^2+^ sensor in triggering neurotransmitter release during stage-II waves.

### Targeted Molecular Perturbation to SACs

To manipulate Syt I molecules in the developing SACs, we developed a molecular perturbation method for the retinal explant culture. We first detected the effects of culture condition on retinal wave dynamics (Fig. S1). We used Ca^2+^ imaging to measure wave-associated Ca^2+^ transients in both acutely isolated retinas and retinal explants after 3–4 day *in vitro* culture. The characteristics of wave-associated Ca^2+^ transients were not significantly changed after *in vitro* culture (Fig. S1A–D). Moreover, both acutely isolated retinas and retinal explants demonstrated similar pairwise correlations over distance for spontaneous Ca^2+^ transients, although acutely isolated retinas demonstrated more correlated waves over longer distances (>400 µm) (Fig. S1E). These data suggest that retinal networks may remain healthy and functional after 3–4 day *in vitro* culture.

**Figure 2:**
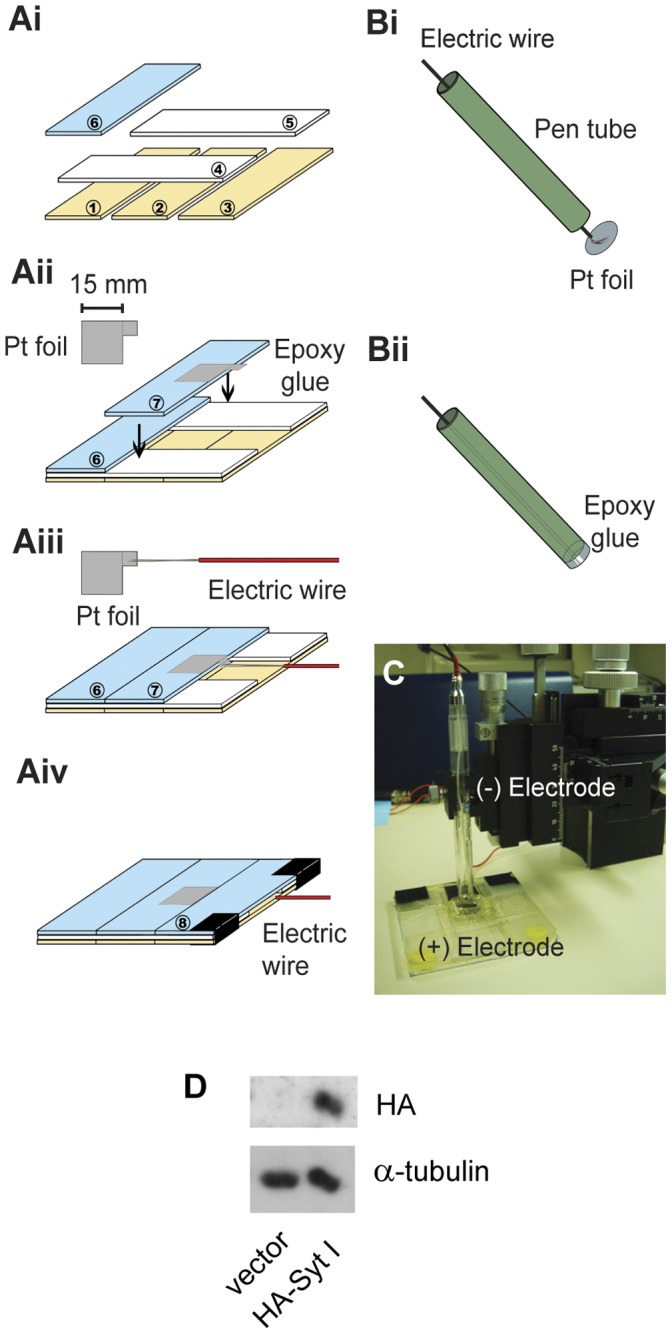
Gene transfer into whole-mount retinas by the homemade electroporation device. A–B. Preparation of platinum (Pt) electrodes. Ai. The arrangement of the six slides for the (+) electrode base. The colors of slides represent the slide arrangement in different layers. The numbers in the circles indicate the corresponding sequence to arrange slides. Aii. The Pt foil (15×15 mm with one extra 5×5 mm-overhanging square) was aligned to one side of the 7^th^ slide with the overhanging square left out. Epoxy glue was applied to attach the Pt foil onto the slide and connect all the slides together. Aiii. The wire was soldered to the edge of the overhanging Pt square. Aiv. The last (8^th^) slide was glued onto the (+) electrode base. Bi–ii. The arrangement of the (−) electrode. A pen tube (green) with a diameter of 10 mm was used to attach the same-sized Pt foil by epoxy glue. The electric wire was inserted through the pen tube and soldered onto the Pt foil. C. The setup for electroporation in a horizontal configuration. The retinal explant was placed in a well [with dimensions 12 (length) ×12 (width) ×3 (height) mm] on the (+) electrode. The (−) electrode made contact with solution above the well and covered the retinal explant. The distance between the (+) and (−) electrodes was adjusted by a micromanipulator that held the (−) electrode. D. The P1 rat retinas were transfected with pCMV-HA (vector) or pCMV-HA-Syt I (HA-Syt I) with this electroporation device. The retinal explants were incubated for 72 hr to allow gene expression. Cellular proteins were solubilized and subjected to SDS-PAGE and Western blot analysis with antibodies indicated on the right (HA or α-tubulin). Only the retinas transfected with HA-Syt I displayed the HA signal with a size of ∼65 kD, which corresponded to the molecular weight of Syt I. Data shown were representative blots from 3 different experiments.

**Figure 3:**
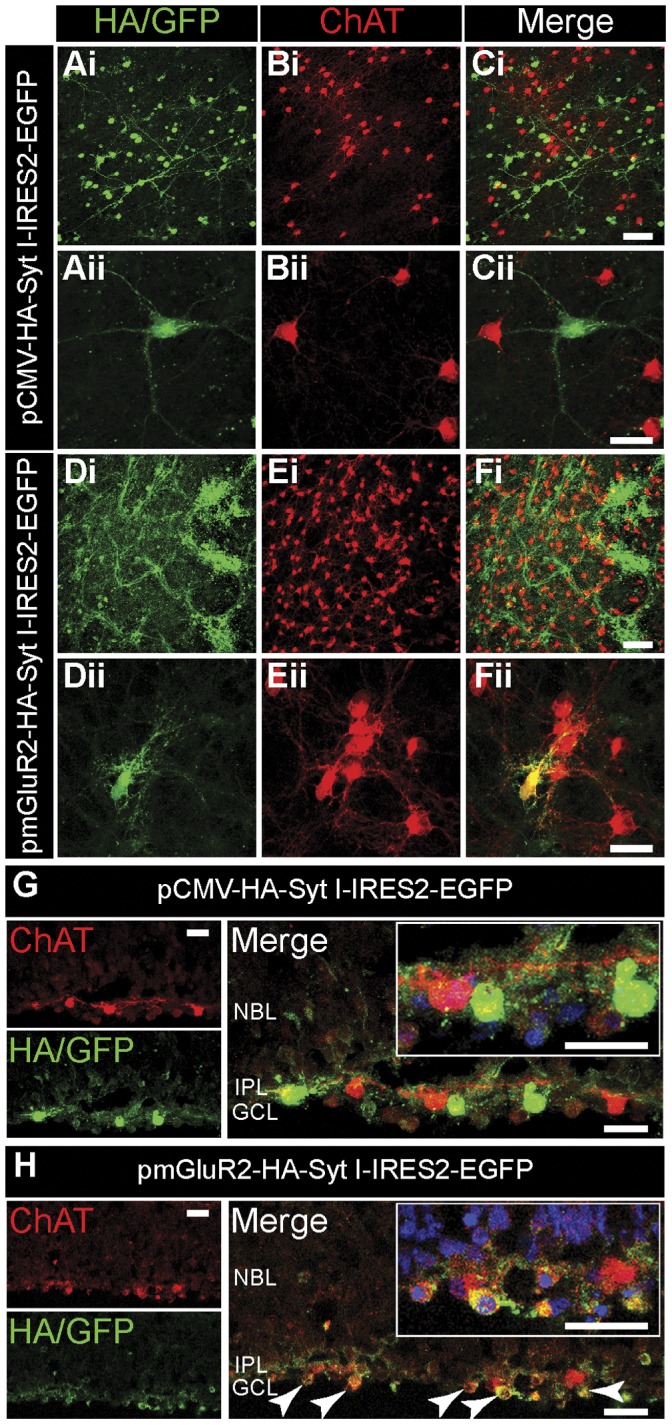
The mGluR2 promoter drives gene expression selectively in SACs. Whole-mount retinas from P0–P2 rats were double-labeled with HA (green) and ChAT (red) after 72 hr of transfection. Ai–Ci. Whole-mount retinas transfected with pCMV-HA-Syt I-IRES2-EGFP. Ai. Labeled with HA. Bi. Labeled with ChAT. Ci. Merged images of Ai and Bi. Scale bar, 50 µm. EGFP, enhanced green fluorescent protein. Aii–Cii. High magnification view of whole-mount retinas transfected with pCMV-HA-Syt I-IRES2-EGFP. Aii. Labeled with HA. Bii. Labeled with ChAT. Cii. Merged images of Aii and Bii. Scale bar, 25 µm. Di–Fi. Whole-mount retinas transfected with pmGluR2-HA-Syt I-IRES2-EGFP. Di. Labeled with HA. Ei. Labeled with ChAT. Fi. Merged images of Di and Ei. Scale bar, 50 µm. Dii-Fii. High magnification view of whole-mount retinas transfected with pmGluR2-HA-Syt I-IRES2-EGFP. Dii. Labeled with HA. Eii. Labeled with ChAT. Fii. Merged images of Dii and Eii. Scale bar, 25 µm. G–H. Retinal cross-sections transfected with either pCMV-HA-Syt I-IRES2-EGFP (G), or pmGluR2-HA-Syt I-IRES2-EGFP (H). The SACs and IPL were stained for ChAT (red). The transfected cells were stained for HA/GFP (green). The cell nuclei were stained with DAPI (blue). *Right,* Merged images (yellow). The transfected SACs were indicated by arrows. *Insets* showed the higher magnification of merged images. Scale bars, 25 µm.

We constructed platinum electrodes to electroporate genes of interest into P0–P2 whole-mount retinas (Fig. 2A–B). This design achieved gene delivery within two parallel electrodes (Fig. 2C). Western blot analysis verified ectopic expression of HA-Syt I in the developing retina by this electroporation strategy (Fig. 2D). This method achieved homogenous expression across the entire retina (Fig. 3ACDF) without altering the major characteristics of retinal waves, such as the frequency, duration, and amplitude of wave-associated Ca^2+^ transients (Table 1). In addition, within 4 days after transfection, retinal waves were reliably blocked by the nAChR antagonist, dihydro-β-erythroidine (DHβE) (10–20 µM) [33], [34]. These results suggest that transfected retinas, which we cultured *in vitro* for 3–4 days after transfection, still generate stage-II waves mediated by cholinergic transmission with the same essential properties. Moreover, no significant differences in wave-associated Ca^2+^ transients were observed in the retinas transfected either on P0 or P1/2 (Table 1). Therefore, in the subsequent Ca^2+^ imaging experiments, we utilized P0–P2 transfected retinas with 3–4 day *in vitro* culture for studying the mechanisms mediating stage-II waves.

**Table 1:** Comparison of wave characteristics following transfection.

	*Wave Frequency* *(No. min^−1^)*	*Wave Duration* *(sec)*	*Wave Amplitude* *(% ΔF/F)*	*# Retinas*
**Intact control**	0.80±0.14	17.21±1.22	2.61±0.51	7
**Transfection on P0**	0.79±0.09	19.19±1.40	2.47±0.25	15
**Transfection on P1/P2**	0.66±0.08	17.95±0.97	2.37±0.21	15

Retinal waves were measured from whole-mount retinal explant cultures after transfection with the DNA plasmid (pmGluR2-IRES2EGFP). Intact control was P0–P2 retinas in the same culture condition (DIV 3–4) without transfection. No significant differences were found among all groups. For wave frequency, *p* = 0.26 (Kruskal-Wallis method with Dunn *post-hoc* test); for wave duration, *p* = 0.58 (One-way ANOVA with Student-Newman *post-hoc* test); for wave amplitude, *p* = 0.87 (One-way ANOVA with Student-Newman *post-hoc* test).

To manipulate neurotransmitter release from SACs during stage-II waves, we aimed to transfect SACs with Syt I mutants with the weakened Ca^2+^ binding in the C2 domains. Since the metabotropic glutamate receptor type II (mGluR2) promoter has been shown to target SACs specifically [7], [35], [36], [37], we placed our Syt constructs under the control of the mGluR2 promoter to manipulate Syt I specifically in SACs. To verify the effectiveness of our transfection strategy, we compared the specificity of the mGluR2 promoter with the ubiquitous cytomegalovirus (CMV) promoter by immnunofluorescence (Fig. 3). With the CMV promoter (Fig. 3A–C), the HA/EGFP immunoreactivity was scarcely colocalized with ChAT (the SAC marker), but mostly appeared in relatively round and large retinal neurons (∼20 µm), likely RGCs. In contrast, with the mGluR2 promoter (Fig. 3D–F), some HA/EGFP immunoreactivity was colocalized with ChAT (Fig. 3Fii, yellow), suggesting that gene expression was efficiently targeted to the SAC somata. The HA/EGFP immunoreactivity appeared in fibers originating from relatively small cells (∼5 µm), similar to SACs (Fig. 3Dii). This pattern is consistent with the expression pattern of endogenous Syt I in the IPL (Fig. 1), justifying that the mGluR2 promoter can target gene expression to SACs and the IPL.

The immunoreactivities from retinal cross-sections were used to evaluate our transfection in SACs. The HA/EGFP immunoreactivity represented all transfected cells (the green cells in Fig. 3GH), whereas ChAT immunoreactivity (in red) indicated SACs. Therefore the transfected SACs would display yellow color (Fig. 3GH). We first determined our transfection efficiency in SACs, which was calculated as the number of cells with yellow signal divided by the number of cells with red signal (DAPI signal indicated nuclei for cell count). The transfection efficiency with the mGluR2 promoter was 57.14±4.56% (mean ± S.E.M., N = 6 sections), compared to 30.25±3.45% (mean ± S.E.M., N = 4 sections) with the CMV promoter.

We then calculated the promoter specificity, which was defined as the number of cells with yellow signal divided by the number of cells with green signal (DAPI signal indicated nuclei for cell count) (Fig. 3GH, *right insets*). With the mGluR2 promoter, the promoter specificity for SACs was 83.87±1.61% (mean ± S.E.M, N = 9 sections), compared to 8.79±1.22% (mean ± S.E.M.) with the CMV promoter (N = 6 sections). Thus, the mGluR2 promoter achieved a significantly higher SAC specificity compared to the CMV promoter (*p*<0.001, two-tailed unpaired *t*-test). This indicates that the mGluR2 promoter can be used to target SACs for molecular perturbation in the developing rat retina.

### Ca^2+^ Transient Frequency is Reduced by Weakened Ca^2+^ Binding to C2A or C2B of Syt I

**Figure 4:**
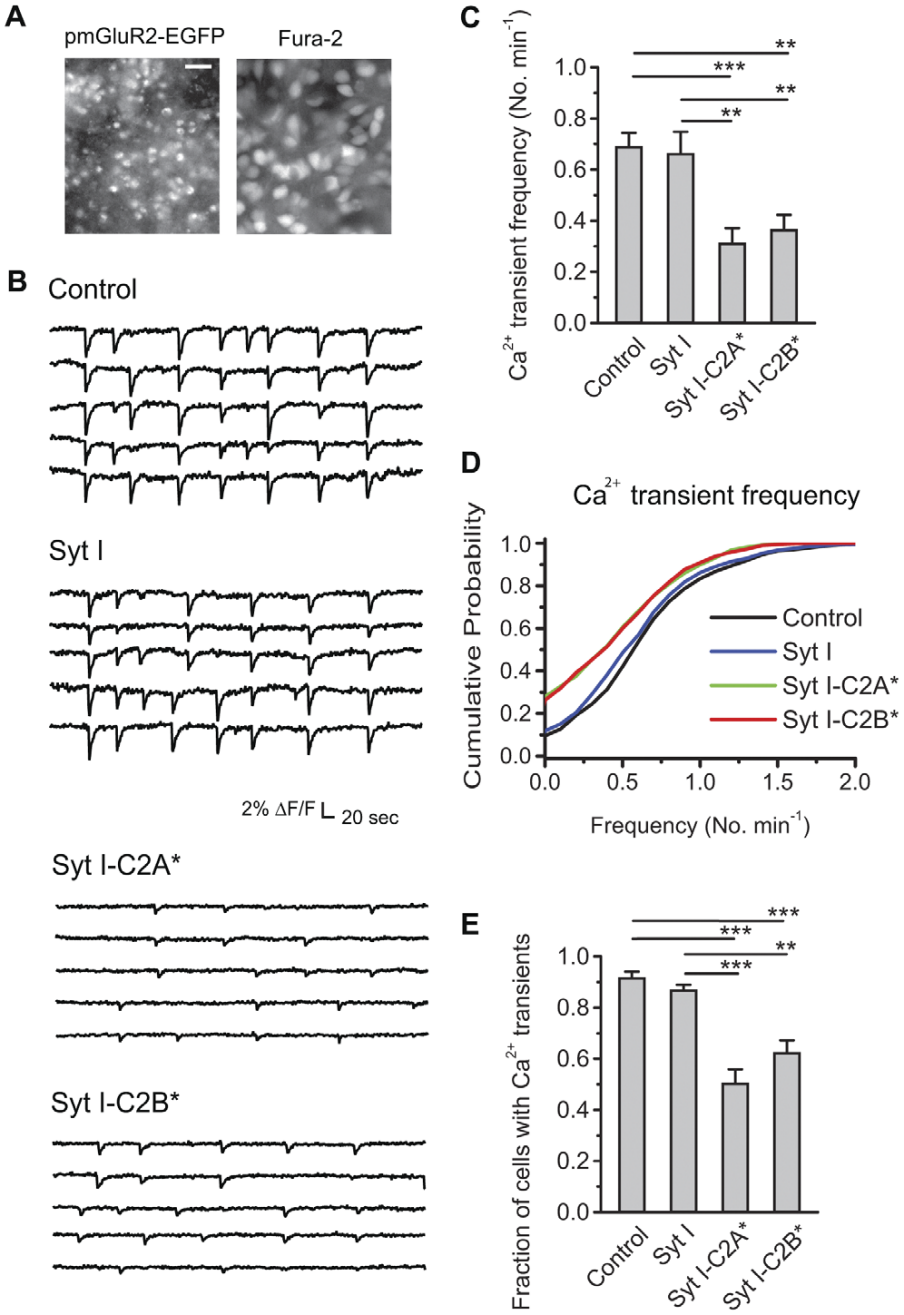
Ca^2+^ transient frequency is reduced by weakened Ca^2+^ binding to the C2AB domains of Syt I. A. *Left,* The expression pattern of pmGluR2-IRES2EGFP in transfected whole-amount retinas. *Right,* The RGC layer labeled with the Ca^2+^ indicator fura-2 to measure the wave-associated Ca^2+^ transients after transfection. Scale bar for both panels, 25 µm. B. Spontaneous, correlated Ca^2+^ transients were monitored from randomly selected cells in the RGC layer. The example traces of fluorescent changes over time showed the Ca^2+^ transients in the nearby cells from one imaged region. Retinas were transfected with pmGluR2-IRES2EGFP (Control), pmGluR2-IRES2EGFP-Syt I (Syt I), pmGluR2-IRES2EGFP-Syt I-D230S (Syt I-C2A*), or pmGluR2-IRES2EGFP-Syt I-D363N (Syt I-C2B*). C. Summary of Ca^2+^ transient frequency after transfection. Data were from 23–39 transfected retinas and 7–15 pups (about 50% data in each group from the retinas transfected on P0 with DIV 3–4). (**p<0.01; ***p<0.001; One-Way ANOVA with *post*-*hoc* Student-Newman-Keuls test.) D. Distributions of cumulative probability for Ca^2+^ transient frequency from individual cells. Data were from 1196–1489 cells out of the same data sets in C. E. Summary of fraction of cells with Ca^2+^ transients in one imaged region after transfection. Data were from 32–58 transfected regions out of the same data sets in C. (**p<0.01; ***p<0.001; Kruskal-Wallis method followed by *post*-*hoc* Dunn test.).

To study the roles of Syt I C2 domains in retinal waves, we used this established electroporation strategy to introduce Syt I mutants with weakened Ca^2+^-binding C2 domains. With the aid of the mGluR2 promoter, transfected retinal explants reliably demonstrated EGFP fluorescence in small cells (∼5 µm), compared to relatively large RGCs (∼20 µm) stained by the Ca^2+^ indicator fura-2 (Fig. 4A). Moreover, the mGluR2 promoter-driven EGFP was localized to the deeper region underneath the RGC layer. The EGFP expression pattern was essentially the same among all transfection groups, suggesting that transfection efficiency was comparable in all groups.

Weakened Ca^2+^ binding to the C2A or C2B domain differentially controls the modes of exocytosis [18], [21]. In particular, D230S confers a Ca^2+^-binding site mutation in the C2A domain of Syt I (designated Syt I-C2A*), whereas D363N confers a Ca^2+^-binding site mutation in the C2B domain of Syt I (designated Syt I-C2B*). Each mutated residue abolishes the first ligand of the third loop in the Ca^2+^-binding pocket of the respective Syt C2 domain [14]. To determine the effects of these Syt I mutants on the Ca^2+^ transient frequency in the developing rat retina, live Ca^2+^ imaging was conducted in the retinal explants after transfection. Spontaneous, correlated Ca^2+^ transients in individual cells revealed retinal waves in the RGC layer of explants transfected with control vector, wild-type Syt I, Syt I-C2A* or Syt I-C2B* (Fig. 4B). To quantify the effects of different transfections on Ca^2+^ transient frequency, we employed two independent measurements, including the change in Ca^2+^ transient frequency (Fig. 4CD) and the fraction of cells displaying Ca^2+^ transients (Fig. 4E).

First, to detect the change in Ca^2+^ transient frequency, the mean frequency was determined from a number of transfected retinas, thereby cancelling the possible transfection variance across retinas or cells. Wild-type Syt I left the mean frequency of Ca^2+^ transients identical to that seen in control retinas (Fig. 4C, mean ± S.E.M. in the number of Ca^2+^ transients per minute: 0.69±0.02 for control, N = 39 retinas; 0.66±0.08 for Syt I, N = 26 retinas; *p*>0.05). In contrast, both Syt I mutants reduced the mean frequency of Ca^2+^ transients by 2-fold compared to wild-type Syt I (Fig. 4B and 4C, mean ± S.E.M. in the number of Ca^2+^ transients per minute: 0.31±0.06 for Syt I-C2A*, N = 23 retinas; 0.36±0.06 for Syt I-C2B*, N = 25 retinas). Thus, weakening Ca^2+^ binding to either the C2A or C2B domain of Syt I reduced Ca^2+^ transient frequency. In addition, the cumulative probability for Ca^2+^ transient frequency was constructed from all recorded cells for control, Syt I, Syt I-C2A* and Syt I-C2B* (Fig. 4D; 1196 cells for control, 1279 cells for Syt I, 1235 cells for Syt I-C2A*, and 1489 cells for Syt I-C2B*). Only 10% of control and Syt I-transfected cells did not display Ca^2+^ transients during the imaging period. In contrast, about 30% of Syt I-C2A* and Syt I-C2B* cells did not display Ca^2+^ transients during the imaging period. The median wave frequency (the frequency at 50% of cumulative probability, in the number of Ca^2+^ transients per minute) was 0.58 for control, 0.55 for Syt I, 0.35 for Syt I-C2A*, and 0.35 for Syt I-C2B* (Fig. 4D), similar to the results of mean Ca^2+^ transient frequency from transfected retinas (Fig. 4C). The curves of cumulative probability for Ca^2+^ transient frequency were left-shifted by Syt I-C2A* and Syt I-C2B*, suggesting that the majority of cells transfected with Syt I-C2A* and Syt I-C2B* display Ca^2+^ transients less frequently compared to control and wild-type Syt I. Thus, weakening Ca^2+^ binding to either C2 domain reduced wave occurrence in most cells.

Second, to determine whether Syt I mutants change the fraction of cells participating in waves, we calculated the fraction of cells with Ca^2+^ transients in one imaged region (i.e. N_w_/49, where N_w_ was the number of cells displaying Ca^2+^ transients during the imaging period and 49 was the number of selected cells in one imaged region). The mean fraction of cells with Ca^2+^ transients in control was indistinguishable from intact, untransfected culture retinas (mean ± S.E.M. in the fraction of cells with Ca^2+^ transients: 0.91±0.03 for intact, N = 10 regions; 0.92±0.02 for control, N = 58 regions; *p*>0.05). Wild-type Syt I left the fraction of cells with Ca^2+^ transients identical to that seen in control cells (Fig. 4D, mean ± S.E.M. in the fraction of cells with Ca^2+^ transients: 0.87±0.02 for Syt I, N = 32 regions; *p*>0.05). The mean fraction of cells with Ca^2+^ transients was significantly reduced in both Syt I-C2A* and Syt I-C2B* compared to control and wild-type Syt I (mean ± S.E.M. in the fraction of cells with Ca^2+^ transients: 0.51±0.06 for Syt I-C2A*, N = 40 regions; 0.62±0.05 for Syt I-C2B*, N = 41 regions). Hence, weakened Ca^2+^ binding to either C2 domain in Syt I reduced the fraction of cells with Ca^2+^ transients. With fewer cells displaying Ca^2+^ transients and less frequent Ca^2+^ transients in most cells, these results suggest that weakened Ca^2+^ binding to the C2AB domains of Syt I may reduce the frequency of stage-II waves.

### Wave-Associated Ca^2+^ Transients are Dampened by Weakened Ca^2+^ Binding to C2A or C2B of Syt I

**Figure 5:**
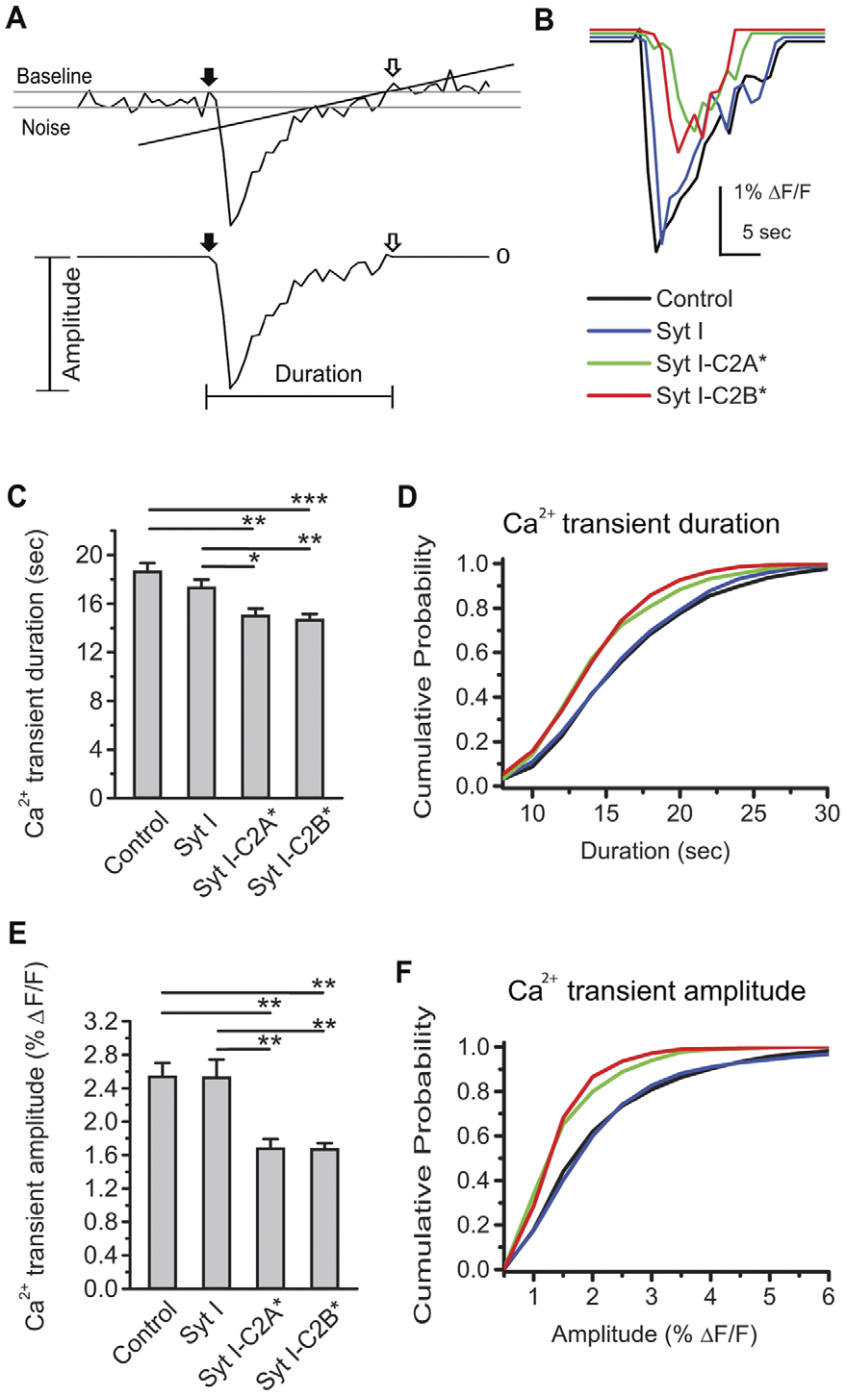
Ca^2+^ transient size is reduced by weakened Ca^2+^ binding to the C2AB domains of Syt I. A. *Top,* Definitions of Ca^2+^ transient duration and amplitude. The peaks of Ca^2+^ transients were automatically picked by Igor Pro according to the criteria in Materials and Methods. The RMS noise (Noise) was measured from the trace between 30 sec prior to the peak and 50 sec following the peak. The starting point (x_0_, y_0_) (indicated by solid arrows) was defined by the latest time point where the first derivative was zero (i.e. dy/dx  = 0, where y was the fluorescence changes in % ΔF/F and x was the recording time in sec) before the peak. To define the end point (x′, y′) (indicated by open arrows), a line was drawn to connect the time points where the trace of Ca^2+^ transients returned to within the RMS noise of the baseline, with the minimal fluctuation of fluorescence between the starting and end points (i.e. y′−y_0_ = minimum). *Bottom,* The Ca^2+^ transient duration was the interval between the starting and end points. The Ca^2+^ transient amplitude was the fluorescence change from the baseline to peak. B. Representative individual Ca^2+^ transients from retinas transfected with Control (black), Syt I (blue), Syt I-C2A* (green), or Syt I-C2B* (red). C. Summary of Ca^2+^ transient duration for each group. Data were from 22–39 transfected retinas and 7–15 pups (about 50% data in each group from the retinas transfected on P0 with DIV 3–4). (*p<0.05; **p<0.01; ***p<0.001; Kruskal-Wallis method followed by *post*-*hoc* Dunn test.) D. Distributions of cumulative probability for Ca^2+^ transient duration from individual cells. Data were from 860–1106 cells out of the same data sets in C. E. Summary of Ca^2+^ transient amplitude after transfection. Data sets were the same as in C. (**p<0.01; Kruskal-Wallis method followed by *post*-*hoc* Dunn test.) F. Distributions of cumulative probability for Ca^2+^ transient amplitude from individual cells. Data sets were the same as in D.

Retinal waves occur with a periodicity on the order of minutes. Spontaneous Ca^2+^ transients associated with waves can last for tens of seconds following relatively brief spontaneous depolarizations [3]. To examine if the wave patterns are changed by Syt I mutants, we measured the duration and amplitude of single Ca^2+^ transients in an unbiased way (Fig. 5A, see Materials and Methods for details). The representative Ca^2+^ transients were shown in Fig. 5B. Both Syt I-C2A* and Syt I-C2B* reduced Ca^2+^ transient size compared to wild-type Syt I and control, including a decrease in Ca^2+^ transient duration and amplitude, and this impression was confirmed by quantitative analysis.

To quantify the effects of Syt I mutants on Ca^2+^ transient duration, we compared the mean durations from different transfection groups. Both Syt I-C2A* and Syt I-C2B* reduced Ca^2+^ transient duration compared to wild-type Syt I and control (Fig. 5C, mean ± S.E.M. in sec: 18.6±0.7 for control, N = 39 retinas; 17.3±0.7 for Syt I, N = 26 retinas; 15.0±0.6 for Syt I-C2A*, N = 22 retinas; 14.6±0.5 for Syt I-C2B*, N  = 23 retinas). Wild-type Syt I did not change the Ca^2+^ transient duration compared to control (*p*>0.05). Moreover, cumulative probability distributions were constructed to get the median Ca^2+^ transient durations (the duration at 50% of cumulative probability; 15.2 sec for control, 15.2 sec for Syt I, 13.4 sec for Syt I-C2A*, and 13.4 sec for Syt I-C2B*) (Fig. 5D), and the differences in these values were consistent with the differences in Ca^2+^ transient duration across retinas (Fig. 5C). The curves of cumulative probability for Ca^2+^ transient duration were left-shifted in Syt I-C2A* and Syt I-C2B*, suggesting that the majority of Syt I-C2A* and Syt I-C2B* cells had briefer Ca^2+^ transients compared to control and wild-type Syt I (Fig. 5D). Thus, weakened Ca^2+^ binding to either the C2A or C2B domain may reduce the duration of wave-associated Ca^2+^ transients.

To quantify the effects of Syt I mutants on Ca^2+^ transient amplitude, we compared the mean amplitude from different transfection groups. Similar to the effects on Ca^2+^ transient duration, wild-type Syt I did not change the Ca^2+^ transient amplitude compared to control (*p*>0.05). Both Syt I-C2A* and Syt I-C2B* significantly reduced Ca^2+^ transient amplitude compared to wild-type Syt I and control (Fig. 5E, mean ± S.E.M. in % ΔF/F: 2.54±0.16 for control, N  = 39 retinas; 2.53±0.21 for Syt I, N = 26 retinas; 1.68±0.11 for Syt I-C2A*, N = 22 retinas; 1.67±0.7 for Syt I-C2B*, N = 23 retinas). Moreover, the median Ca^2+^ transient amplitudes (the amplitude at 50% of cumulative probability, in % ΔF/F: 1.8 for control, 1.8 for Syt I, 1.25 for Syt I-C2A*, and 1.25 for Syt I-C2B*) were also reduced in Syt I-C2A* and Syt I-C2B* (Fig. 5F), similar to the results of the mean Ca^2+^ transient amplitude (Fig. 5E). The curves of cumulative probability for Ca^2+^ transient amplitude were also left-shifted in Syt I-C2A* and Syt I-C2B*, suggesting that the majority of Syt I-C2A* and Syt I-C2B* cells had relatively small amplitude of Ca^2+^ transients compared to control and wild-type Syt I (Fig. 5F). Together, these results show that weakened Ca^2+^ binding to either the C2A or C2B domain in Syt I dampened the wave-associated Ca^2+^ transients.

### Spatial Correlation of Retinal Waves is not Altered by Weakened Ca^2+^ Binding to C2AB of Syt I

**Figure 6:**
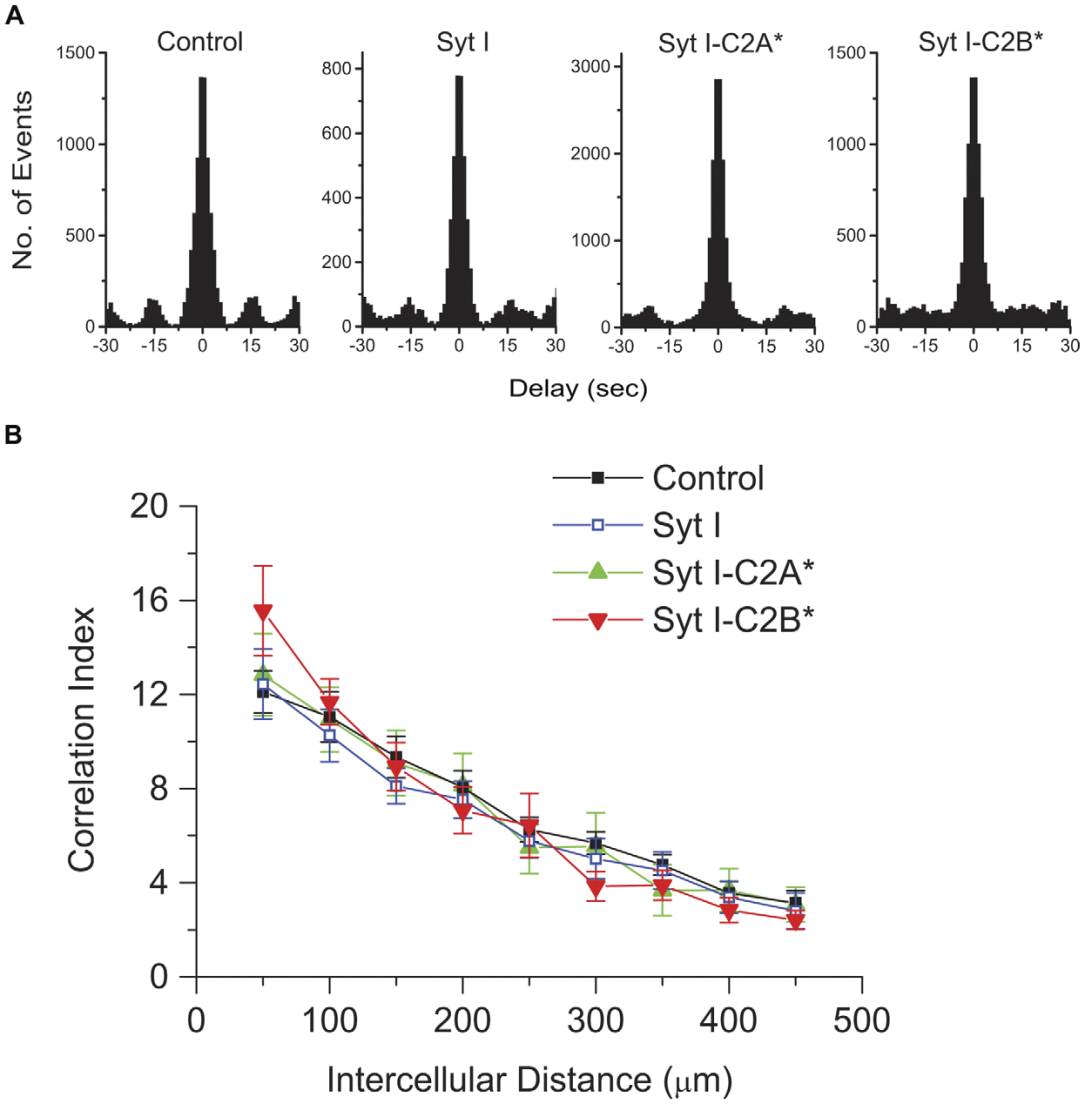
Pairwise correlation is not altered by weakened Ca^2+^ binding to the C2AB domain of Syt I. A. Representative pairwise cross-correlograms of 49 cells in one imaged region from the transfected retinas. Each event was a Ca^2+^ transient in an individual cell. Delay times were the time differences between Ca^2+^ transient peaks in cell pairs. Note that all correlograms for control, Syt I, Syt I-C2A* and Syt I-C2B* were very similar with sharp peaks at t = 0 sec (see Table 2 for statistics). The oscillatory behavior of the correlograms beyond | t | > ∼7.5 sec might reflect the bursting behavior of the distant cells. Bin widths for all correlograms were 1 sec. B. Pairwise C.I. values as a function of intercellular distance for control, Syt I, Syt I-C2A* and Syt I-C2B*. Data points were averages of medians within the 50 µm bins from 17–23 transfected retinas (about 50% data in each group from the retinas transfected on P0 with DIV 3–4). The error bars were the standard errors of the C.I. values in each bin.

Both Syt I-C2A* and Syt I-C2B* dampened wave-associated Ca^2+^ transients, but it is unknown if these mutants also affect the spatial properties of stage-II waves (i.e. the correlated propagation over distance). To assess wave correlation structure across cells, we computed cross-correlograms over cell pairs [38], [39] within imaged regions (340×460 µm). The representative cross-correlograms for control, Syt I, Syt I-C2A* and Syt I-C2B* were shown in Fig. 6A. The sharp peaks at t = 0 sec in all the correlograms for cell pairs (Fig. 6A; Data summarized in Table 2) indicated that the waves were well correlated in the imaged region. Syt I-C2B* had more oscillatory behavior outside of t = 0 sec compared to all other groups. We thus suspected that waves in distant cell pairs lose their synchrony in the Syt I-C2B* transfected retina.

**Table 2:** Comparison of wave correlograms for Syt I and its C2AB mutants.

	*Gaussian SD (sec)*	*# Regions*	*# Retinas*
**Control**	1.762±0.093	55	38
**Syt I**	1.596±0.121	32	24
Syt I-C2A*	1.532±0.128	24	16
Syt I-C2B*	1.717±0.114	32	22

Retinal waves were measured from cultured whole-mount retinas expressing Syt I or its mutants (Syt I-C2A* and Syt I-C2B*) with the mGluR2 promoter. Control was transfected with pmGluR2-IRES2EGFP. Wave correlograms were constructed from 49 cell pairs in one imaged region and fitted to a Gaussian equation to yield the Gaussian mean and standard deviation (SD). No significant differences were found in Gaussian mean (0.00±0.00 sec) among all groups. For Gaussian SD, *p* = 0.6225 (One-Way ANOVA with Student-Newman *post-hoc* test).

To further quantify the strength of the correlations, the correlation index (C.I.) [38], [40] was computed and plotted as a function of intercellular distance (Fig. 6B). In all groups, the C.I. values were highest for nearby cells, and then decreased as a function of distance, consistent with the persistence of propagating waves. Although we suspected that Syt I-C2B* conferred the highest value of maximal C.I. and these C.I. values dropped off more quickly (Fig. 6B), the C.I. values at any given distance were not significantly different among all groups (P>0.05). Thus, weakened Ca^2+^ binding to the C2AB domain of Syt I may not alter the spatial correlation structure of stage-II waves.

## Discussion

In this study, we showed that Syt I is strongly expressed in cholinergic neurons (SACs) and synapses (the IPL) in retinas exhibiting stage-II waves. We further established a molecular perturbation method targeted to presynaptic SACs. By weakening Ca^2+^ binding in the C2AB domains of Syt I, we tested the effects of presynaptic perturbation on wave-associated Ca^2+^ transients. Our Ca^2+^ imaging results provide the first demonstration that Ca^2+^ binding to C2A or C2B of Syt I regulates the temporal properties of stage-II waves. In contrast, Ca^2+^ binding to C2AB in Syt I has relatively minor effects on the spatial properties of stage-II retinal waves. These results suggest that the C2AB domains of Syt I regulate the dynamics of patterned spontaneous activity during the development of neural circuits.

### Presynaptic Syt I in Regulating Patterned Spontaneous Activity

While the importance of Syt I in synaptic transmission has been recognized for a couple of years [15], its significance in regulating large-scale, repetitive synaptic circuit activity has yet been reported. In our study, we found that Syt I regulates the dynamics of wave-associated Ca^2+^ transients via Ca^2+^ binding to its C2AB domains. Together with the previous findings [5], [31], our present study suggest that the Ca^2+^ oscillations in SACs [5] may cause Ca^2+^ binding to its sensor Syt I to trigger exocytosis [31] during stage-II waves. Hence, modulating fusion machinery in SACs alone may regulate wave dynamics.

Developing SACs corelease ACh and GABA in a Ca^2+^-dependent manner [5]. Both ACh and GABA are stored in synaptic vesicles, thereby sharing the common releasing mechanism. Note that GABA also exerts the excitatory effects during stage-II waves in rats [37]. Thus, advanced work is required to identify whether the effects we observed in this study may be due to the changes in ACh or GABA release, or both. Moreover, although it is a challenging work to directly measure exocytosis in SACs, how release from SACs is influenced by Ca^2+^ binding to Syt I warrants further investigation, especially in the form of kiss-and-run or full fusion.

Manipulations that alter the spatiotemporal properties of stage-II waves result in defective retinogeniculate and retinocollicular projections [40], [41], [42], [43], [44]. Although the significance of retinal waves has gained recognition, previous studies of retinal wave generation focused only on “postsynaptic” loci, such as the nAChR during stage-II waves [45], [46], [47]. Our study first demonstrates that alterations in “presynaptic” fusion machinery can also regulate stage-II waves, thereby providing alternative targets for the manipulation of retinal waves. The previous studies have identified that stage-II waves are present from P0 until P9 in rats [33], [37]. Our preliminary results showed that Syt I began to be expressed in the outer plexiform layer after P9 (unpublished data), implying that Syt I may also involve in regulating stage-III waves in rats. More work for the effects of various exocytotic molecules (including SNARE proteins and Syt isoforms) on the spatiotemporal pattern of retinal waves will thus enhance our ability to control developmental transitions in the visual system.

### The Roles of Syt I C2AB Domains in Shaping Patterned Spontaneous Activity

The roles of the C2AB domains in Syt I have been intensively investigated at the molecular and functional levels. Biochemical analysis showed that both Syt I-C2A* and Syt I-C2B* greatly reduce their binding to t-SNARE heterodimers (corresponding to target proteins on plasma membrane) and PS-containing liposomes (corresponding to vesicle membrane constituent) [15], [19], [21]. However, only Syt I-C2B* reduces binding to PIP2-containing liposomes (corresponding to vesicle and plasma membrane constituent) [15], [19], [21], [48] and homo-oligomerization with other Syt I [49]. Moreover, studies of fusion pore kinetics revealed that Syt I-C2A* switches the exocytosis mode from full fusion to small kiss-and-run with no dilation step [18]. The long duration of these small kiss-and-run events has been proposed to make neurotransmitter release very slow, possibly elevating ambient extracellular neurotransmitter concentrations [18]. In contrast, Syt I-C2B* abolishes both exocytosis modes, thereby leaving brief kiss-and-run events which can hardly release neurotransmitter to support synaptic responses [21]. All these molecular and functional findings suggest that the C2AB domains exert significant effects on exocytosis by engaging distinct effector interactions. Thus, our findings may be directly linked to the regulation of fusion machinery in SAC release.

Alternative interpretation for the effects of Syt I mutations on wave dynamics is that overexpression of Syt I-C2A* and Syt I-C2B* might alter cytoplasmic Ca^2+^ dynamics in SACs and thus reduce exocytosis. However, several *in vitro* and *in vivo* studies have demonstrated that overexpression of Syt I with Ca^2+^-binding site mutations can not alter whole-cell Ca^2+^ current or Ca^2+^ dynamics [15], [19], [20], [23], [24], implying that the effects of presynaptic perturbations in the present study cannot be solely attributed to the changes in Ca^2+^ channels or dynamics in SACs.

The spatial properties of stage-II waves arise from a single SAC that innervates multiple RGCs and other SACs [7], resulting in correlated Ca^2+^ transients in nearby RGCs. We observed that Syt I-C2A* and Syt I-C2B* dampen the temporal properties of stage-II waves (Fig. 4–5), while these two mutants cannot alter the spatial properties of stage-II waves (Fig. 6). One explanation for this observation is that in transfected retinas, a few SAC terminals may still retain the capacity for ACh release contributed from endogenous wild-type Syt I to support the propagation of stage-II waves. Thus, the C2AB domain of Syt I cannot significantly influence the spatial correlation structure of retinal waves. Alternatively, the C2B domain of Syt I may slightly affect pairwise correlations, which is beyond the limitation of instrumentation.

### Future Directions

A previous study revealed that Ca^2+^ influx into RGCs during long-duration waves can induce a significant increase in cAMP/PKA activity [33]. Thus, the RGCs with long-duration waves may lead to activity-dependent gene expression via the cAMP/PKA/CREB pathway, which has been shown to be essential for visual circuit development [50]. In our study, both Syt I-C2A* and Syt I-C2B* significantly reduce Ca^2+^ transient duration compared to Syt I (Fig. 5), suggesting that downstream gene activation in neighboring RGCs may be reduced by presynaptic Syt I-C2A* and Syt I-C2B*. How gene expression patterns in postsynaptic RGCs are impacted by expressing presynaptic Syt I, Syt I-C2A* and Syt I-C2B* is an important topic for future investigation.

Retinal waves propagate through the developing retina, inducing similar burst patterns in the thalamus and visual cortex to form sensory loops [3]. Although similar burst patterns occur in the synapses of these sensory loops, the identities and roles of the Syt isoforms in each synapse have yet to be determined. In particular, the etiology of schizophrenia and autism has been attributed to a developmental disorder of “neural connectivity” in the formation of sensory maps, especially in the thalamus [51], [52], [53], [54]. Therefore, it is important to identify and characterize the roles of exocytotic molecules in developing neural circuits. This will provide new insight and contribute to the development of novel therapeutic methods for human psychiatric diseases.

## Materials and Methods

### Molecular Biology

The metabotropic glutamate receptor subtype II (mGluR2) promoter was used for SAC-specific expression [35], [36], [37]. First, the cDNAs encoding Syt I were subcloned into the pCMV-IRES2EGFP (Clontech #6029-1). The mutants Syt I-C2A* (D230S) and Syt I-C2B* (D363N) were prepared by the modified sequential PCR procedure [55] with the pCMV-IRES2EGFP encoding wild-type Syt I as template [18]. Second, the CMV promoter was replaced with the mGluR2 promoter. The resultant vector pmGluR2-IRES2EGFP allowed gene expression targeted to SACs under the control of the mGluR2 promoter. Enhanced green fluorescent protein (EGFP) was translated from the same strand of coding mRNA driven by the internal ribosome entry site (IRES), so the EGFP fluorescence was used to identify transfected SACs for Ca^2+^ imaging (Fig. 4A). For Western analysis (Fig. 2D), the pCMV-HA-Syt I was prepared by subcloning the Syt I cDNA into pCMV-HA vector (Clontech #631604). For immunofluorescence (Fig. 3), the pCMV-HA-Syt I-IRES2EGFP and pmGluR2-HA-Syt I-IRES2EGFP were prepared by subcloning the IRES2EGFP fragment into pCMV-HA-Syt I and pmGluR2-HA-Syt I, respectively. The pmGluR2-HA-Syt I was previously acquired from pCMV-HA-Syt I by replacing the CMV with the mGluR2 promoter. All constructs were confirmed by sequencing. The DNA plasmids were amplified (Qiagen) and dissolved in Tris buffer (10 mM, pH 7.6) for transfection.

### Ethics Statement and Animals

This study was carried out in strict accordance with the recommendations in the Guide for the Care and Use of Laboratory Animals of the National Institutes of Health. The protocol for all animal experiments was approved by the Institutional Animal Care and Use Committee of National Taiwan University (Permit Number: 97-27 and 99-82). Neonatal (P0–P2) Sprague-Dawley rats (BioLASCO, Taiwan) were used in the study. All rat pups were housed with their own mothers in the individually ventilated cages with well-controlled conditions (12∶12 light/dark cycle with light on at 7 AM; 20±1°C) and *ad libitum* access to food and water. Rat pups (P0–P2) were deeply anesthetized with ice before decapitation with all efforts to minimize suffering.

### Retinal Explant Culture

The retina was isolated and cut into thirds in dissection buffer [1X HBSS (GIBCO), 10 mM HEPES and 0.35 g/L NaHCO_3_, pH 7.35]. The retinal pieces were attached onto nitrocellulose membranes (Millipore) with the RGC layer facing up. Retinal explants were cultured at 35°C in a 5% CO_2_ humidified incubator, and daily supplied with fresh Retinal Serum-Free Culture Medium (SFCM-A), containing Neurobasal-A (GIBCO #10888), 0.6% Glucose, 2 mM L-Glutamine (Sigma #G6392), 1X B-27 (GIBCO #17504-044), 10 mM HEPES, 1 mM Sodium Pyruvate (GIBCO #11360-070), 2.5 µg/mL Insulin (Sigma #I1882), 100 µg/mL Penicillin/100 µnits/mL Streptomycin (GIBCO #15140-122), and 6 µM Forskolin [33].

### Transient Transfection

DNA plasmids (200 ng/µL in dissection buffer) encoding the proteins of interest were transiently transfected into cultured retinal explants. About 400 µL of DNA-containing buffer was used for transfection of each retinal explant (80 µg DNA per transfection). Before electroporation, the retinal explants were incubated in the DNA-containing buffer at room temperature (RT) for 10 min to enhance transfection efficiency. The homemade platinum electrodes with a horizontal configuration were connected to the electroporator (27 V; 50 ms of pulse duration; 2 pulses at 1 sec-interval; BTX ECM830 electroporator, Harvard Apparatus). The horizontal distance of the electrode gap was 4 mm to yield an electric strength of 6.75 V/mm. One retinal explant with the RGC layer facing up (toward the (−) electrode in Fig. 2C) was placed within the electrode gap filled with the DNA-containing buffer. The electric pulses were applied with the parameters as mentioned above. Right after electroporation, the retinal explants were immediately placed in the cultures and maintained for 60–96 hr (3–4 days *in vitro*, DIV 3–4).

### Immunofluorescence

For whole-mount retina staining, the transfected retinal explants were placed on poly-lysine-coated slides, fixed with 4% paraformaldehyde (PFA) at RT for 30 min, and washed with phosphate buffered saline [PBS, with 136.89 NaCl, 2.68 KCl, 10.14 Na_2_HPO_4_, and 1.76 KH_2_PO_4_ in (mM)] for 1 hr. After fixation, the explants were blocked at RT for 1 hr in 3% donkey-serum blocking solution (DBS), containing 3% donkey serum (Jackson Lab #017000121), 0.5% Triton X-100, and 0.1% sodium azide in PBS. The explants were first incubated with primary antibodies in 1% DBS [goat polyclonal anti-ChAT (1∶200; Millipore #AB144P) and mouse anti-HA (1∶800; Covance #MMS-101P)] at RT for 2 days, and then washed with PBS. Secondary antibodies in 1% DBS [donkey-anti-goat IgG conjugated with Alexa Fluor 568 (1∶400; Invitrogen #A11057) and donkey-anti-mouse IgG conjugated with Alexa Fluor 488 (1∶400; Invitrogen #A21202)] were further added at RT for 1 day, and then washed out with PBS. The anti-fade reagent Fluoromount G (Electron Microscopy Sciences #17984-25) was added to retinal explants on the slides. The slides were sealed by coverslips. Fluorescent images were acquired by confocal microscopy (Leica TCS SP5 spectral, Germany).

For retinal cross-section staining, transfected retinal explants were fixed with 4% PFA at 4°C overnight. After fixation, the explants were cryoprotected in 30% sucrose at 4°C for 1 day and preserved in Tissue-Tek optical cutting temperature (O.C.T.) gel (Sakura Finetech #4583). Retinal cross-sections (16 µm) were prepared with a cryostat (Leica CM1850), placed on poly-lysine-coated slides, washed with PBS, and blocked at RT for 1 hr in 3% DBS. The retinal sections were incubated with primary antibodies at 4°C overnight, washed with PBS, further incubated with secondary antibodies at RT for 1 hr, and washed with PBS again. The samples were finally stained with DAPI (Sigma #D9542) at RT for 10 min. The slides and immunofluorescence images were acquired as mentioned above. Quantification of colocalization was determined with MetaMorph software (Version 7.5, Molecular Devices).

### Western Analysis

The P0–P2 rat retinal explants transfected with pCMV-HA or pCMV-HA-Syt I were spun down in dissection buffer at ∼2,000 *g* at 4°C for 5 min, and stored at −80°C. The transfected retinal explants were lyzed with a homogenizer (Branson Sonifier S-250D) in the ice-cold RIPA lysis buffer (1% NP-40, 150 mM NaCl, 1 mM EDTA, 1 mM phenylmethylsulfonyl fluoride, 1 mM sodium orthovanadate, 1 mM sodium fluoride, and 50 mM HEPES, pH 7.4, supplemented with protease inhibitors). After sonication, samples were centrifuged at 18,000 *g* at 4°C for 10 min and protein concentration was determined by Bradford assay (Bio-Rad #500-0006). Protein in the cellular lysate (20 µg) was electrophoresed through standard 10% Laemmli SDS polyacrylamide gels, transferred to polyvinylidene fluoride membranes, and probed with appropriate antibodies. Membranes were blocked for 1 hr in 5% non-fat milk in TBST (100 mM Tris-HCl, pH 7.5, 150 mM NaCl, and 0.1% Tween-20) and then incubated with primary antibodies [mouse anti-HA (Covance #MMS-101P) and mouse anti-α-tubulin (Sigma #T5168)] at 4°C overnight. Membranes were washed three times with TBST and then incubated with secondary antibody in 5% milk in TBST at RT for 1 hr. Membranes were washed three times with TBST, and the signals were visualized by enhanced chemiluminescence followed by autoradiography.

### Live Ca^2+^ Imaging

Retinal explants were transferred to forskolin-free SFCM-A overnight before imaging. Prior to Ca^2+^ imaging, retinal explants were placed in the forskolin-free SFCM-A containing 10 µM fura-2-AM (Molecular Probes #F1221), 0.02% pluronic acid (Molecular Probes #P3000MP), and 1% DMSO for 30–60 min. During Ca^2+^ imaging experiments, retinal explants were perfused continuously with artificial cerebrospinal fluid (ACSF) containing (in mM): 119 NaCl, 26.2 NaHCO_3_, 2.5 KCl, 1.0 K_2_HPO_4_, 1.3 MgCl_2_, 2.5 CaCl_2_, and 11 D-glucose bubbled with 95% O_2_/5% CO_2_. The temperature of ACSF was maintained at 30°C with a TC-344B controller (Warner Instruments). Live Ca^2+^ imaging was performed on an upright Olympus BX51WI, using a 20X water immersion objective (Olympus). The transfected regions in the retinal explants were identified by EGFP fluorescence (Ex 470/Em 525, Chroma #41017). The fura-2 fluorescence was excited at 380 nm (Chroma #D380xv2) via a xenon arc lamp (300 Watts, DG-4, Sutter Instrument) and a 455DCLP dichotic mirror (Chroma). The fura-2 emission fluorescence was collected at 510 nm (Chroma #D510/40 m) and captured by a CCD camera (CoolSNAP HQ2, Photometrics). To measure spontaneous Ca^2+^ transients associated with retinal waves, the fura-2 fluorescence images were continuously acquired at 1 sec-intervals for 10 min (totally 601 time frames), with 100–150 msec exposure times and two as binning number. One or two transfected regions from each retinal explant were chosen for 10-min recordings. Digitized imaging data were subsequently processed by MetaMorph software. Further data analysis was conducted by Igor Pro (WaveMetrics), Excel and Orgin 5 or 8 (OriginLab).

### Data Analysis

The Ca^2+^ imaging data for each transfection group were acquired by the following procedures. First, 49 RGCs were randomly selected from the 7×7 divisions in one imaged region (340×460 µm) using MetaMorph. The wave data from single RGCs were acquired from the fluorescence changes across all the time frames, previously background subtracted for each frame. The fluorescence signals were further corrected for photobleaching by using Igor Pro to set the baseline. Second, to analyze wave characteristics (frequency/duration/amplitude) in an unbiased way, an Igor procedure was written in this laboratory to automatically pick the wave peaks, with their fluorescence intensity 2-fold greater than the RMS noise (∼0.25% ΔF/F). The definitions of the duration and amplitude for individual waves are presented in Fig. 5A. Third, all wave data were averaged from each cell, averaged across 49 cells out of one imaged region, and then averaged from two imaged regions out of one retina. Finally, the mean wave data for each group were averaged from all retinas transfected with the same gene. Distributions of cumulative probability were constructed from single-cell wave data for the same transfection group.

Correlation of Ca^2+^ waves between nearby cells was calculated from pairs of RGCs in the same imaged region separated by various distances (10–500 µm). The positions of individual cells were calculated using MetaMorph. The intercellular distance was taken as the shortest distance between selected cells. The lag times of the waves in these two nearby cells were acquired by individually subtracting from the presenting time of every wave in the paired cells. All the subtracted times were collected for each cell pair to construct cross-correlograms from one imaged region (Fig. 6A) [39]. In addition, the lag times of the waves in these two nearby cells were used to calculate the correlation index (C.I.) (Fig. 6B) [38], [40]. The C.I. is a measure of the likelihood relative to chance of a pair of neurons firing together within a given interval (Δ*t*  = 3 sec), as given by the following equation:
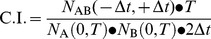

*N*_AB_ is the wave number for which cell B fires with a time ± Δ*t* from cell A, *N*_A_ and *N*_B_ are the total numbers of waves fired by cells A and B, respectively, during the total recording time (both *N*_A_ and *N*_B_ ≥ 3 in 10 min), *T* is total recording time (600 sec), and Δ*t* is the correlation time interval (3 sec). The cell pairs from one imaged region were grouped according to their intercellular distances with a bin width of 50 µm. The median C.I. values were computed from each distance group out of the same imaged region. The averaged C.I. values were computed from the same distance group out of many imaged regions for each transfection group, and then plotted against the upper limit of intercellular distance for each distance group (Fig. 6B).

### Statistics

All data were presented as mean ± S.E.M. Differences between means of different groups (≥3 groups) were evaluated for statistical significance with One-way ANOVA followed by *post*-*hoc* Student-Newman-Keuls test for the parametric method, or Kruskal-Wallis method followed by *post*-*hoc* Dunn test for the nonparametric method (InStat 3, GraphPad).

## Supporting Information

Figure S1 **Comparison of wave-associated Ca^2+^ transients in acutely isolated retinas (Acute) and retinal explants after 3–4 day** ***in vitro*** **culture (Culture).** (A) Ca^2+^ transient frequency. (B) Fraction of cells with Ca^2+^ transients. (C) Ca^2+^ transient duration. (D) Ca^2+^ transient amplitude. (E) Pairwise C.I. values as a function of intercellular distance. The methods to measure the properties of Ca^2+^ transients were described in Fig. 4, 5, 6. Data were from 8 acutely isolated retinas (P2) and 7 retinal explants (dissected on P0-2 with DIV 3–4). (*p<0.05, unpaired *t*-test for comparison at a given distance; n.s., not significantly different from acutely isolated retinas)
(TIF)
