# Supplementary figures and images for: Synaptotagmin I Regulates Patterned Spontaneous Activity in the Developing Rat Retina via Calcium Binding to the C2AB Domains
Source: PLoS One. 2012 Oct 16;7(10):e47465. doi: 10.1371/journal.pone.0047465 (PMC3472990; doi:10.1371/journal.pone.0047465)

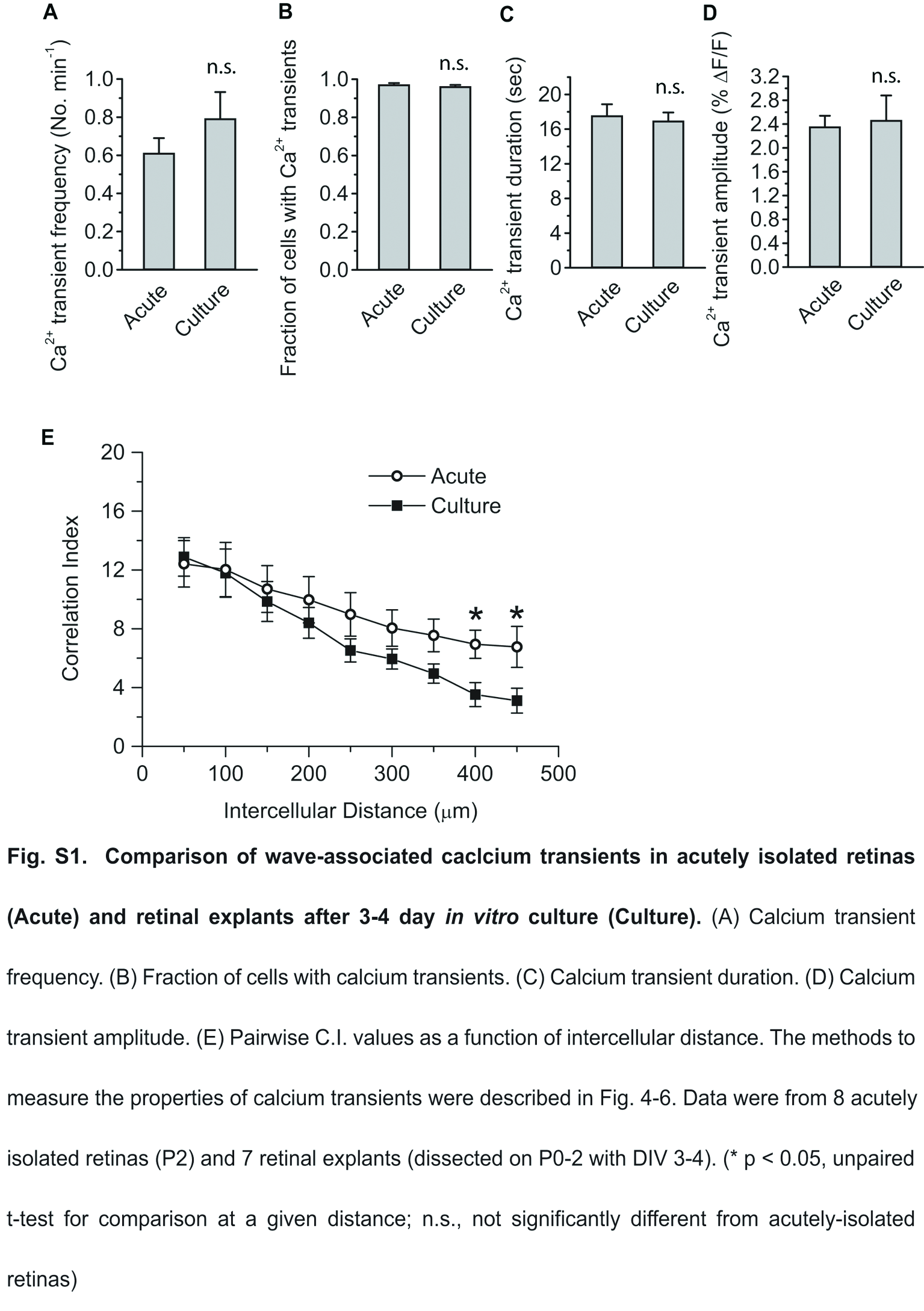

Supplement: Figure S1 — Comparison of wave-associated Ca2+ transients in acutely isolated retinas (Acute) and retinal explants after 3–4 day in vitro culture (Culture). (A) Ca2+ transient frequency. (B) Fraction of cells with Ca2+ transients. (C) Ca2+ transient duration. (D) Ca2+ transient amplitude. (E) Pairwise C.I. values as a function of intercellular distance. The methods to measure the properties of Ca2+ transients were described in Fig. 4, 5, 6. Data were from 8 acutely isolated retinas (P2) and 7 retinal explants (dissected on P0-2 with DIV 3–4). (*p<0.05, unpaired t-test for comparison at a given distance; n.s., not significantly different from acutely isolated retinas) (TIF) [file pone.0047465.s001.tif]
